# A pragmatic implementation research study for *In Our DNA SC*: a protocol to identify multi-level factors that support the implementation of a population-wide genomic screening initiative in diverse populations

**DOI:** 10.1186/s43058-022-00286-2

**Published:** 2022-04-28

**Authors:** Caitlin G. Allen, Daniel P. Judge, Elissa Levin, Katherine Sterba, Kelly Hunt, Paula S. Ramos, Cathy Melvin, Karen Wager, Kenneth Catchpole, Catherine Clinton, Marvella Ford, Lori L. McMahon, Leslie Lenert

**Affiliations:** 1 Department of Public Health Sciences, Medical University of South Carolina, Charleston, SC USA; 2 Division of Cardiology, Medical University of South Carolina, Charleston, SC USA; 3 Clinical & Policy, Helix, San Mateo, CA USA; 4 Department of Medicine, Department of Public Health Sciences, Medical University of South Carolina, Charleston, SC USA; 5 Department of Healthcare Leadership and Management, College of Health Professions, Medical University of South Carolina, Charleston, SC USA; 6 Anesthesia & Perioperative Medicine, Medical University of South Carolina, Charleston, SC USA; 7 Hollings Cancer Center, Medical University of South Carolina, Charleston, SC USA; 8 Office of Vice President for Research, Department of Neuroscience, Medical University of South Carolina, Charleston, SC USA; 9 Biomedical Informatics Center, Medical University of South Carolina, Charleston, SC USA

**Keywords:** Implementation science, Population-based genomic screening, Health services research

## Abstract

**Background:**

In 2021, the Medical University of South Carolina (MUSC) partnered with Helix, a population genetic testing company, to offer population-wide genomic screening for Centers for Disease Control and Preventions’ Tier 1 conditions of hereditary breast and ovarian cancer, Lynch syndrome, and familial hypercholesterolemia to 100,000 individuals in South Carolina. We developed an implementation science protocol to study the multi-level factors that influence the successful implementation of the *In Our DNA SC* initiative.

**Methods:**

We will use a convergent parallel mixed-methods study design to evaluate the implementation of planned strategies and associated outcomes for *In Our DNA SC.* Aims focus on monitoring participation to ensure engagement of diverse populations, assessing contextual factors that influence implementation in community and clinical settings, describing the implementation team’s facilitators and barriers, and tracking program adaptations. We report details about each data collection tool and analyses planned, including surveys, interview guides, and tracking logs to capture and code work group meetings, adaptations, and technical assistance needs.

**Discussion:**

The goal of *In Our DNA SC* is to provide population-level screening for actionable genetic conditions and to foster ongoing translational research. The use of implementation science can help better understand how to support the success of *In Our DNA SC*, identify barriers and facilitators to program implementation, and can ensure the sustainability of population-level genetic testing. The model-based components of our implementation science protocol can support the identification of best practices to streamline the expansion of similar population genomics programs at other institutions

Contributions to the literature

- Expands implementation science methodologies to a new field of precision health
- Builds on efforts to incorporate a health equity lens to implementation science protocols
- Offers an example of the use of the implementation research logic model to develop a parallel mixed methods study to assess program implementation

## Background

Genetic information can help personalize disease prevention and early detection efforts, leading to better clinical and population health outcomes [1, 2]. The Centers for Disease Control and Prevention (CDC) recognizes three genetic conditions: hereditary breast and ovarian cancer syndrome (HBOC), Lynch syndrome (LS), and familial hypercholesterolemia (FH) as “Tier 1” conditions. A Tier 1 designation indicates that there is sufficient evidence and that established interventions are available to reduce morbidity and mortality of individuals who are identified with these conditions. Combined, Tier 1 conditions affect 1–2% of the US population; however, few know their risk or receive information about their genetic condition at the time of disease diagnosis [3–6].

Screening for Tier 1 conditions has commonly occurred through family history collection with follow-up genetic testing recommended among individuals who have a strong family history. Several barriers exist to collecting family health information that can be used to inform clinical practice, resulting in less than one-third of the population being knowledgeable of their family history [7–11]. In addition to the logistical challenges associated with collecting family health history, this approach may not provide the necessary information to identify individuals at high genetic risk. For example, a recent population-wide genomic screening effort for the BRCA1/2 variant (associated with HBOC) improved the identification of individuals with deleterious variants. Approximately 50% of individuals in these studies did not have a personal or family history that would indicate an increased risk for cancer [3, 12].

The rapidly decreasing cost of genetic sequencing and increased throughput ability have paved the way for a population-based approach to genetic screening [13, 14]. Growing evidence supports population-wide genomic screening for Tier 1 conditions among healthy adults with or without personal or family history [14], including in historically underrepresented populations [15]. In 2018, the Genomics and Population Health Action Collaborative, an ad hoc collaboration with the Roundtable on Genomics and Precision Health at the National Academies of Sciences, Engineering, and Medicine, developed a roadmap for the implementation of population-wide genomic screening programs [16]. This report emphasized the utility of screening for Tier 1 conditions and urged thoughtful implementation with clear strategies to evaluate the impact of these approaches in the context of medicine and society [16]. Other critical considerations for equitable implementation of population-wide genomic screening include educating the genomics/precision health workforce, increasing awareness for the power of genomics/precision health, informing policy decisions, improving data infrastructure and the evidence base, as well as issues related to ethical, legal, and social implications (ELSI) and diversity, equity, and inclusion (DEI) [15].

Despite the increasing accessibility of population-wide genomic screening for Tier 1 conditions in health systems, challenges exist to scaling up these efforts, including engaging large multidisciplinary teams of researchers and clinicians, ensuring participant’s understanding of genetic information, equitable access and participation in population testing, and sustainability of programs [16–19]. Strategies from implementation science can help identify successful implementation strategies, support the integration of genomic information captured by population screening in health systems, and provide lessons learned about fundamental elements of population-wide genomic screening programs that influence support delivery of similar programs across diverse settings [20, 21].

### Description of *In Our DNA SC* partnership and conceptual model

In 2021, the Medical University of South Carolina (MUSC) partnered with Helix, a leading population genomics company, to offer population-level genetic testing to participants in South Carolina. This partnership, called *In Our DNA SC*, is designed to provide genetic testing for up to 100,000 people by 2025, initially reporting pathogenic or likely pathogenic variants in CDC Tier 1 conditions (*APOB*, *BRCA1*, *BRCA2*, *EPCAM*, *LDLR*, *LDLRAP1*, *MLH1*, *MSH2*, *MSH6*, *PCSK9*, *PMS2*). *In Our DNA SC* involves recruitment, consent, collection of a saliva sample, processing the sample through the Helix laboratory, receipt of results (positive and negative results), and genetic counseling for participants who receive positive results. Current recruitment approaches for *In Our DNA SC* include an automated recruitment message sent through the electronic health record patient portal to individuals who have a clinical appointment at a participating clinic. Eligible individuals receive two additional messages via the patient portal and those who express interest but do not consent receive a follow-up text message and phone call from a research coordinator. Additional planned recruitment strategies include self-schedule visits with study team members and walk-up participation at community events.

The program involves several work groups that are co-led by MUSC and Helix to oversee essential elements of the program, including marketing and communications, data and technology to recruit participants and return results, clinical operations and staff training, clinical services re-use, and genomic research enablement to support future use of data among MUSC researchers. *In Our DNA SC* also established an IMPACTeam (IMPlementAtion sCience for *In Our DNA SC* Team) to create a strategy to assess implementation, service, and clinical outcomes related to the program using principles of implementation science.

The IMPACTeam uses the Implementation Research Logic Model (IRLM) to provide a structure for describing how determinants, implementation strategies, and mechanisms of change influence outcomes related to *In Our DNA SC* [22]. The IRLM comprehensively documents all potential determinants of implementation among stakeholder groups and links them to corresponding levels of outcomes to be assessed throughout the program. Our conceptual framework incorporates determinants from the Consolidated Framework for Implementation Research (CFIR) among participants/community members, implementation teams within MUSC, and providers and staff at clinical sites. CFIR was designed as a comprehensive framework to describe multi-level implementation determinants in five domains: intervention characteristics, inner setting, outer setting, characteristics of the individual, and process [23].

*In Our DNA SC* currently plans a multi-phased implementation approach, which includes a pilot phase (implementation at 10 clinical sites), followed by institutional expansion across all clinical sites at MUSC and community expansion to patients not currently affiliated with MUSC. Implementation strategies associated with each phase include planning (e.g., developing charters and formal implementation blueprints, identifying, and preparing champions), quality management (e.g., tracking systems, centralizing technical assistance), education (e.g., distribution of technical assistance materials, conducting ongoing training), and restructuring to support scale-up. Associated mechanisms will also be evaluated to assess programmatic outcomes that occur among participants (e.g., raising awareness, improving reach), implementation teams (e.g., clarifying roles and ensuring accountability), and providers and staff at implementation sites (e.g., facilitating uptake at clinical sites, having supportive staff that can answer participant questions) [24].

**Fig. 1:**
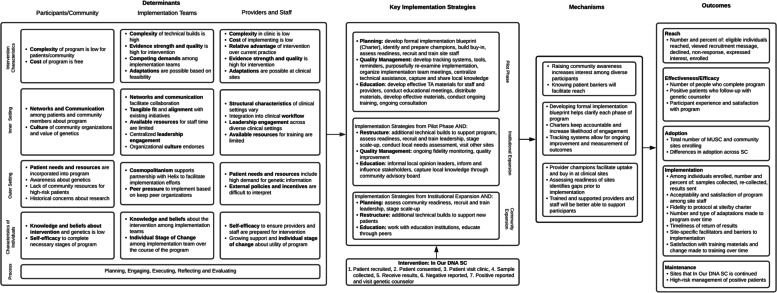
*In Our DNA SC* Implementation Science Conceptual Model

We will evaluate implementation outcomes using the Reach, Effectiveness, Adoption, Implementation, Maintenance (RE-AIM) framework [25]. These outcomes will be tracked continuously throughout the program, and we will leverage findings about these outcomes to inform each phase of *In Our DNA SC* expansion, as well as modifications to implementation strategies and adaptations to the program itself (see Fig. 1).

### Objectives and aims

The goal of this protocol is to describe the implementation process and strategies to support *In Our DNA SC.* Incorporating implementation and evaluation theories and methods as part of the program’s efforts from the beginning will help facilitate this population-wide genomic screening initiative at MUSC, enhance access to genomic services among populations not traditionally engaged, as well as support initiatives across other health systems. This approach is guided by four aims (see Table 1).

**Table 1:** Summary of implementation aims

Aim	Quantitative analyses			Qualitative analyses
	Analysis unit and method	Primary predictors	Outcomes (RE-AIM)	
**Aim 1:** Monitor participation in *In Our DNA SC,* identify, and assess factors associated with participant engagement	**Analysis unit:** Individual **Analysis method:** All outcomes assessed cross-sectional (weekly reports to leadership) and longitudinally	*Demographics:* • Age • Sex • Race • Ethnicity *Collection site*	*Reach:* • # of eligible individuals reached • # viewed recruitment message • # declined • # non-response • # expressed interest • # enrolled *Implementation:* Among those enrolled in *In Our DNA SC*: • # of samples collected • # Re-collected • # Results sent • Timeliness of return of results *Effectiveness/efficacy:* • Number of people who complete *In Our DNA SC* • Positive patients who follow-up with genetic counselor *Maintenance:* • High-risk management of positive patients (number of people visiting genetic counselor who schedule screening)	*Participant interviews* • *Efficacy*: Participant experiences and satisfaction with the program • Rapid analysis initially to provide necessary information • Full, in-depth coding using thematic analysis approach • Participants stratified based on the type of engagement (declined, non-response, enrolled)
**Aim 2:** Assess contextual factors and strategies that may influence adoption and sustainment of *In Our DNA SC* among clinical and community sites and ongoing site-specific needs related to program implementation	**Analysis unit:** Site **Analysis method:** Bivariate analyses and multivariable linear regression to assess influence of predictors on adoption and maintenance	Use of training materials Use of community-facing education materials Implementation site logs (number and type of facilitators and barriers identified) Research coordinator logs (number and type of question) Site readiness survey (AIM, IAM, FIM)	*Adoption:* • Total number of MUSC and community sites enrolling • Differences in adoption across SC *Maintenance:* Sites that *In Our DNA SC* is continued	Implementation Site Logs: Rapid deductive qualtiative analysis
**Aim 3:** Describe facilitators and barriers to implementation and perceptions of *In Our DNA SC* among work group members	**Analysis unit:** Work group, implementation team **Analysis method:** Frequency and percent, mean and standard deviation; cross-sectional and longitudinal	*Work group logs:* Summarize implementation barriers and facilitators by work group and over time *Check-in survey:* Summarize perception of program by work group and over time		Work Group Logs: Rapid deductive qualitative analysis
**Aim 4: **Track adaptations made to *In Our DNA SC* to assess how mechanisms of change impact key programmatic outcomes	**Analysis unit**: Program **Analysis method:** Summary of type of adaptations made	*Implementation:* • Number and type of adaptations made to program over time	*All RE-AIM outcomes* and whether positive, negative, or no impact	N/A

Aim 1: Monitor participation in *In Our DNA SC,* identify, and assess factors associated with participant engagement

Aim 2: Assess contextual factors and strategies that may influence adoption and sustainment of *In Our DNA SC* among clinical and community sites and ongoing site-specific needs related to program implementation

Aim 3: Describe facilitators and barriers to implementation and perceptions of *In Our DNA SC* among work group members

Aim 4: Track adaptations made to *In Our DNA SC* to assess how mechanisms of change impact key programmatic outcomes

## Methods and design

### Overall study design

To achieve our four implementation-focused aims, we will use a convergent parallel mixed-methods study design to assess the planned implementation strategies and the effectiveness of *In Our DNA SC.* This approach will allow us to simultaneously collect quantitative and qualitative data, merge the data, and use the results to provide a comprehensive understanding of the implementation of *In Our DNA SC*. We will use rapid assessments of the implementation strategies (e.g., fidelity checks, training, and technical assistance) to inform changes to the program. Additionally, our design will track planned incidental program adaptations [26]. All aspects of this study have been approved by the Medical University of South Carolina as Exempt, with a Waiver of Consent granted.

**Fig. 2:**
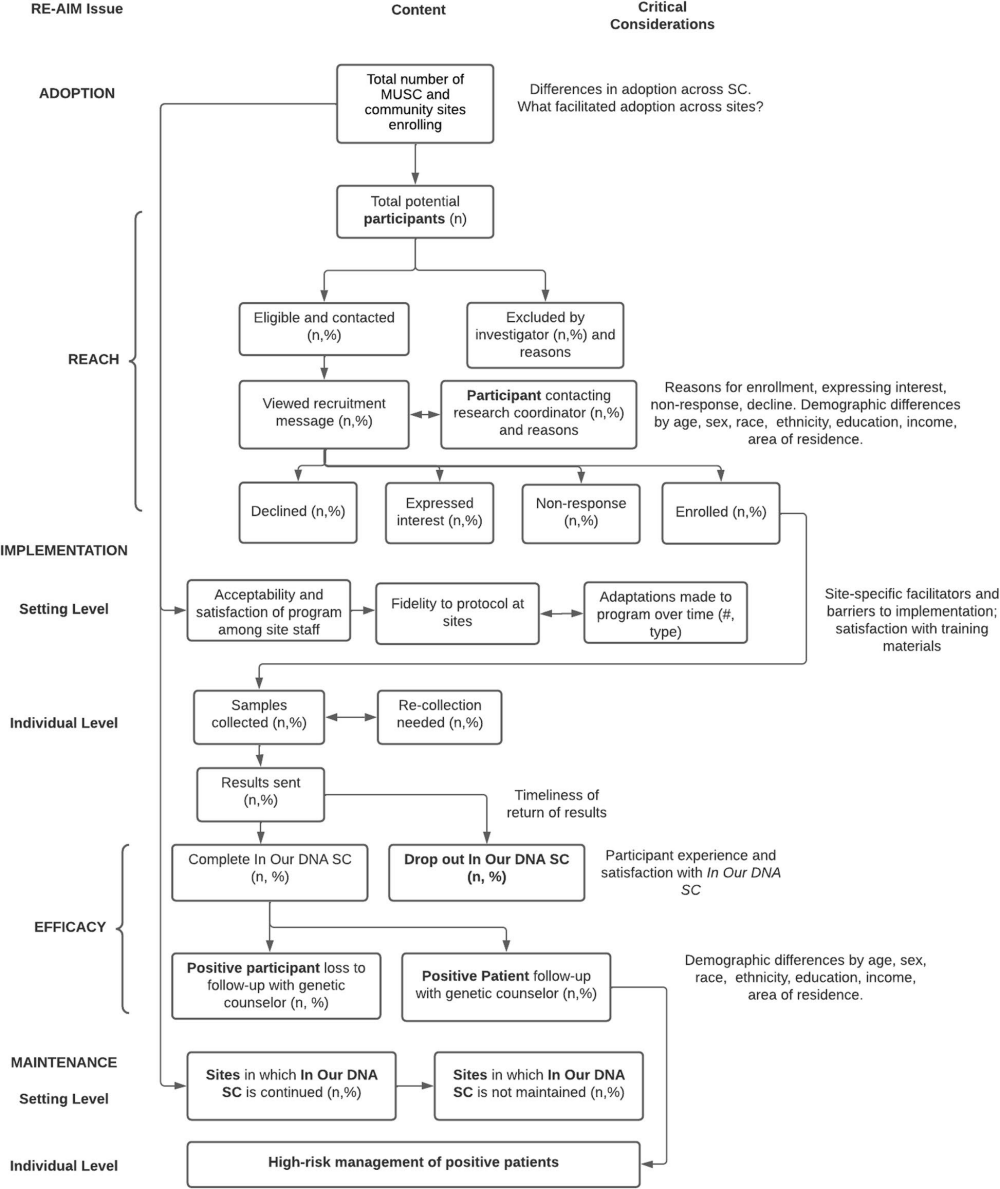
Extended CONSORT Diagram for *In Our DNA SC* Implementation Science

We will use the expanded CONSORT diagram to standardize internal reporting and produce rapid, rigorous, transparent, and relevant information (see Fig. 2) [27, 28].

### Data collection strategies

We currently plan to collect data from ten sources with additional data collection approaches developed, as needed. These strategies include a data dashboard, participant interviews, use of training materials, use of community-facing educational materials, implementation site logs, site readiness surveys, work group logs, adaptation logs, research coordinator logs, and check-in surveys (see Table 2).

**Table 2:** Data collection strategies

Strategy	Description	Frequency	Type of data	Use (aim)
Data dashboard	• Recruitment messages sent • Declined • Non-response • Expressed interest • Enrolled • Samples collected • Re-collection needed • Results sent to Helix • Demographics (age, sex, race, ethnicity, education, income, area of residence)	Ongoing	Quantitative	Aim 1
Participant interviews	Semi-structured interview guide to qualitatively assess the experience of individuals who are part of *In Our DNA SC*	Every 6-months throughout the duration of the program	Qualitative	Aim 1
Use of training materials	• View pre-recorded training materials • Download brochures and handouts	During the training period for sites (pre-implementation)	Quantitative	Aim 2
Use of community-facing educational materials	Review of the utility of community-facing educational materials	Every 6-months throughout the duration of the program	Quantitative	Aim 2
Implementation site logs	Tracking of technical assistance calls with implementation sites; coded with CFIR to track facilitators and barriers	Weekly meetings	Quantitative (open-ended questions)	Aim 1, 2
Site readiness survey	Assessment of readiness for implementation and perceptions of the program using AIM, IAM, and FIM	Pre-implementation	Quantitative (open-ended questions)	Aim 1, 2
Work group logs	Tracking of working group meetings and progress; coded using CFIR to track facilitators and barriers	Weekly meetings	Quantitative (open-ended questions)	Aim 3
Adaptation logs	Tracking of changes made throughout the course of implementation and outcomes associated with change	Ongoing/as adaptations are made	Quantitative (open-ended questions)	Aim 4
Research coordinator logs	Tracking of questions and technical assistance needs from patients, clinicians, providers, implementation teams	Ongoing	Quantitative	Aim 1, 2
Check-in surveys	Periodic surveys sent to work group members to capture the experience and provide barriers and facilitators using RE-AIM framework	Pre-launch of a new phase of the program	Quantitative (open-ended questions)	Aim 3

#### Data dashboard

Monitoring participant recruitment into *In Our DNA SC* and saliva sample collection will take place through a data dashboard in the electronic health record. The data dashboard includes summary information about the total number of recruitment messages sent, declined, non-response, express interest, enrolled, samples collected, sample re-collection (original sample was not sufficient), results sent to Helix, results returned to the participant, number of positive individuals who complete genetic counseling, and number who schedule an additional screening. Data will also be stratified by demographics, based on information available from the electronic health record, including age, sex, race, ethnicity, education, income, and area of residence.

#### Participant interviews

Qualitative data will also be collected to further probe in areas of significant drop off or where there are discrepancies in the anticipated and actual numbers of individuals based on demographic categories. Current areas identified for qualitative investigation include (a) individuals who do not enroll in *In Our DNA SC* either because they decline or review the invitation to participate and take no action, (b) people who participate in *In Our DNA SC* and receive negative results, and (c) people who participate in *In Our DNA SC* and receive positive results. Individuals who are associated with the *In Our DNA SC* study will be invited to participate in brief interviews via phone or email. The interview questions will be guided by a semi-structure interview guide tailored to the type of individual being interviewed. We will focus on recruiting diverse individuals to capture perceptions about genetics specifically addressing DEI challenges with engagement. We will conduct interviews every 6 months throughout the duration of the program (anticipated to be 48 months).

#### Use of training materials

A major aspect to a successful implementation of *In Our DNA SC* involves saliva sample collection. We will assess the contextual factors and strategies that influence how samples are being collected. Training materials about sample collection are available among clinical sites affiliated with MUSC. Data collection about the use of training materials includes tracking the completion of training materials about the study available sites through MyQuest (internal training site) and Horseshoe (internal website). Data will be captured prior to implementation at a clinical site using quantitative methods.

#### Use of community-facing education materials

To help support the engagement of historically underrepresented populations in genomics research, the *In Our DNA SC* program will develop educational materials to be delivered in community settings (e.g., via community health workers and other public health workforces). These materials and planned community expansion. We will assess the impact these materials have on individual’s perceptions and understanding of genetics and the success of community engagement efforts. These efforts will occur alongside a community advisory board.

#### Implementation site logs

Qualitative data will also be by tracking weekly technical assistance calls with sites implementing the *In Our DNA SC* program sites and coding these meetings using CFIR with a focus on executing, participant needs and resources, implementation climate, and leadership engagement [23]. The logs have space to capture open-ended notes about these discussions.

#### Site readiness survey

We will assess readiness for implementation and perceptions of the program using quantitative measures of Acceptability of Intervention Measure (AIM), Intervention Appropriateness Measure (IAM), and Feasibility of Intervention Measure (FIM), and open-ended response options [29]. These surveys will be distributed to identify provider champions and clinical site leads prior to implementation at new clinical and community sites.

#### Work group logs

We designed a REDCap survey to capture notes at each scheduled work group meeting. Information captured includes date of meeting, overview of the topics discussed, and option to save key documents provided during the meeting. Following each work group meeting, the study team will use the REDCap survey to identify which components of CFIR were addressed whether these elements are considered facilitators or barriers [23].

#### Adaptation logs

Adaptation logs gather data about the changes being made to the *In Our DNA SC* program. Adaptation tracking includes a brief description of the adaptation made, whether it was planned or unplanned, when in the program the adaptation was made, what changed (e.g., content, context, training), the nature of the modification (e.g., tailoring, repeating a component of the intervention, changing the order of components of intervention), who initiated (e.g., leadership, specific work group, stakeholder), who the change impacts (e.g., patient, implementation teams), the basis on which the changes were made (e.g., based on summary information, financial incentives), why the change was made (e.g., to increase reach, to improve adoption), the impact of the change (e.g., positive, negative), and long term impact of the change (e.g., increase reach, improve participation by teams, improved ability to deliver intervention successfully) [24, 30–32].

#### Research coordinator logs

Research coordinator logs will capture questions and technical assistance needs from participants, clinicians and providers, and implementation teams. Details about the type of question and whether follow-up is needed are included in the research coordinator log.

#### Check-in surveys

Check-in surveys will be sent to implementation teams (work group members) prior to the launch of a new phase of the program to capture their experience with *In Our DNA SC*. These surveys assess how confident implementation teams are in the status of the program using the RE-AIM framework [25].

### Qualitative data analysis

#### Participant interviews

All interviews will be transcribed and quality controlled. We will initially conduct rapid qualitative analysis to provide necessary information to inform ongoing program development. Rapid qualitative analysis involves developing a templated summary table to extract interview data, including illustrative quotes for each interview. Next, the interview summaries will be consolidated by participant type in a data matrix to capture themes, sub-themes, and supporting quotes. Data from this step will be used to report to program leadership and optimize implementation and expansion [33]. Full, in-depth coding will occur using thematic analysis at program completion.

#### Implementation site logs and work group logs

*In Our DNA SC* operational staff facilitates weekly implementation calls where sites implementing the program gather to discuss questions, concerns, and technical assistance needs. A member of the IMPACTeam team participates in these weekly calls and tracks facilitators and barriers to implementation using a REDCap tracking tool that includes space to take notes about the facilitators and barriers identified and CFIR themes. Initial qualitative analyses are conducted in real-time. Themes from implementation site logs will be reported in summary across sites. Work group themes will be recorded and summarized by work group and in summary across work groups. This rapid deductive approach allows for us to capture information in real-time to help inform the process of implementation [34]. Once coded, the information from the implementation site logs are used as quantitative data in Aim 2 and work group logs are used as quantitative data in Aim 3.

### Quantitative data analysis

Descriptive analyses will be performed prior to conducting statistical analyses for each aim.

#### Aim 1: Monitor participation in In Our DNA SC, identify, and assess factors associated with participant engagement

The primary outcomes of interest for Aim 1 are at the individual level and include reach (total number of eligible people reached based on recruitment messages, declined, non-response, express interest, and enrollment), implementation (number of samples collected, re-collected, and results sent), effectiveness/efficacy (positive patients who complete program, positive patients who follow-up with genetic counselor).

Predictors for the primary data analysis include information collected through the data dashboard described above, including demographics (age, sex, race, ethnicity, education, income, and area of residence).

 We will use simple linear or logistic regression to assess for bivariate associations between all demographic predictors on each outcome of interest. We will also conduct multivariable regression to assess the influence of all demographic characteristics and fixed covariates for each collection site as predictors of each outcome. Data will be reported weekly as well as longitudinally to assess for trends in each outcome over time.

#### Aim 2: Assess contextual factors and strategies that may influence adoption and sustainment of In Our DNA SC among clinical and community sites and ongoing site-specific needs related to program implementation

The primary outcomes of interest include adoption (total number of MUSC and community sites enrolling participants, differences in adoption across sites in South Carolina) and maintenance (sites that continue to promote population-wide genomic screening beyond initial funding period).

Predictors used to assess for outcomes related to Aim 2 include the use of training materials, implementation site logs (number and type of facilitators and barriers identified), research coordinator logs (number and type of questions asked), site readiness (AIM, IAM, FIM), and utility of community-facing materials [29].

All outcomes and predictors are assessed at the site level. We will conduct bivariate analyses using simple linear or logistic regression with outcomes of interest. We will also use multivariable linear regression to assess the influence of all predictors on outcomes of interest at the clinical and community site levels.

#### Aim 3: Describe facilitators and barriers to implementation and perceptions of In Our DNA SC among work group members

We will summarize the implementation barriers and facilitators identified during each work group meeting as part of the work group logs. These will be reported stratified by work group and summarized across all work groups. We will assess changes in facilitators and barriers longitudinally across the program. In addition, we will summarize perceptions of *In Our DNA SC* using findings from the check-in survey. Additionally, we will assess change in work group perceptions longitudinally across the program.

#### Aim 4: Track adaptations made to In Our DNA SC to assess how mechanisms of change impact key programmatic outcomes

The data collected as part of Aim 4 is primarily designed to provide structure to track the rollout of *In Our DNA SC* and to capture the implementation over time. All quantitative data captured through the adaptation tracking logs will be regularly assessed through descriptive analyses. Information from each log will be reported in aggregate (e.g., types of adaptations made, key CFIR facilitators, and barriers identified in work group logs) on an ongoing basis. The longitudinal tracking will allow the team to assess various mediators and moderators of program outcomes. We will develop additional tracking tools as needed over the course of program implementation.

### Integrating quantitative and qualitative methods

Across all aims, data will be integrated to provide rapid feedback to the implementation teams and leadership overseeing *In Our DNA SC*. The IMPACTeam will closely monitor factors that influence DEI and the representation of racial and ethnic minorities in the program. Reported barriers and facilitators will be matched with evidence-based mechanisms of change and implementation strategies to quickly resolve implementation barriers and tailor implementation strategies to be suitable for groups that have historically been excluded from genetic research [30]. We will continue to use this approach of rapid assessment and intervention modification throughout the stages of program implementation and adjust our approaches to monitoring participation as needed.

## Discussion

This implementation research seeks to leverage the existing infrastructure and implementation efforts of *In Our DNA SC*, a population-wide genomic screening initiative to assess key implementation and effectiveness outcomes. Our goal is to continuously provide feedback to support the implementation and sustainability of *In Our DNA SC*, which can enable precision-based clinical engagement of subpopulations who could benefit most. Information gathered during this study will help provide a foundation for additional research needed to support population-wide genomic screening and efforts to integrate routine genetic screening into clinical practice.

A major goal of *In Our DNA SC* is to engage the diverse populations of South Carolina. According to the 2020 US Census Bureau, approximately 69% of South Carolinians identify as White, 27% as Black or African American, 6% as some other race, and 6% as two or more races [35]. Our focus on the inclusion of historically underrepresented and diverse groups, understanding their preferences towards genomic testing, and assessing the role of tailored educational strategies in improving participant engagement, are unique strengths of *In Our DNA SC.* Results from this program are expected to enhance diversity and help mitigate the health disparities and inequities resulting from the lack of diversity in genomics research. Given the emphasis of *In Our DNA SC* in diversity and inclusion of historically underrepresented or marginalized populations, and the legacy of misuse and misapplication of genetic research in certain groups and communities, special attention is being given to ELSI of this initiative to local communities.

We use a pragmatic approach to the collection and analysis of quantitative and qualitative data. For example, we use a rapid qualitative analysis method to initially assess patient interviews. This study captures data in real-time, allowing data to inform the direction of the program and match implementation strategies to key barriers. Furthermore, our approach to studying the program is guided by a strong conceptual framework that incorporates well-recognized implementation science theories of RE-AIM and CFIR [23, 25].

Our planned implementation research activities are not without limitations. The *In Our DNA SC* initiative was designed to be implemented in a dynamic way with various phases of staging and growth. While the general outline of how *In Our DNA SC* will be expanded exists (e.g., pilot phase, on boarding of additional clinical site, and community site expansion), as the program grows, the exact way that each phase is implemented may change. Our study seeks to account for how these changes impact the key outcomes through close tracking using the Adaptation Log; however, additional tools or methodologies and analyses we have not accounted for in the current protocol may be needed to assess the program as it grows. Additionally, our most distal clinical effectiveness outcome is whether a positive individual who is at high risk for HBOC, LS, or FH completes genetic counseling and scheduling recommended follow-up screening. We do not assess for the clinical management and long-term adherence to recommendations for the management of high-risk individuals (e.g., oophorectomy for those identified with HBOC). Additional efforts are needed to track clinical management and cascade screening of identified families. These limitations suggest the need for future efforts to continue building evidence about the clinical utility of population-wide genomic screening.

We have developed a series of research questions and associated data collection tools to assess factors that may influence the implementation of population-wide genomic screening among individuals, clinical staff and providers, community groups, and implementation teams. These efforts focus on ensuring access to this program among populations that have commonly been excluded in genetic research and clinical services. Our pragmatic approach to studying how a population-based effort is implemented in a health system can support the generalization of the lessons from *In Our DNA SC* and identify best practices to streamline the expansion of similar initiatives in other settings.

## Data Availability

N/A. No data have been collected.

## Abbreviations

MUSC: Medical University of South Carolina
HBOC: Hereditary Breast and Ovarian Cancer
LS: Lynch syndrome
FH: Familial hypercholesterolemia
ELSI: Ethical legal and social implications
DEI: Diversity equity and inclusion
